# CHFR negatively regulates SIRT1 activity upon oxidative stress

**DOI:** 10.1038/srep37578

**Published:** 2016-11-24

**Authors:** Myungjin Kim, Young Eun Kwon, Jae Oh Song, Sung Jun Bae, Jae Hong Seol

**Affiliations:** 1 School of Biological Sciences, Research Institute of Basic Sciences, IMCB, Seoul National University, Seoul 08826, Korea

## Abstract

SIRT1, the NAD^+^-dependent protein deacetylase, controls cell-cycle progression and apoptosis by suppressing p53 tumour suppressor. Although SIRT1 is known to be phosphorylated by JNK1 upon oxidative stress and subsequently down-regulated, it still remains elusive how SIRT1 stability and activity are controlled. Here, we have unveiled that CHFR functions as an E3 Ub-ligase of SIRT1, responsible for its proteasomal degradation under oxidative stress conditions. CHFR interacts with and destabilizes SIRT1 by ubiquitylation and subsequent proteolysis. Such CHFR-mediated SIRT1 inhibition leads to the increase of p53 acetylation and its target gene transcription. Notably, CHFR facilitates SIRT1 destabilization when SIRT1 is phosphorylated by JNK1 upon oxidative stress, followed by prominent apoptotic cell death. Meanwhile, JNK inhibitor prevents SIRT1 phosphorylation, leading to elevated SIRT1 protein levels even in the presence of H_2_O_2_. Taken together, our results indicate that CHFR plays a crucial role in the cellular stress response pathway by controlling the stability and function of SIRT1.

CHFR (checkpoint protein with FHA and RING finger domains) is a RING-type E3 ubiquitin (Ub)-ligase, which regulates numerous important cellular proteins, i.e., PLK1, Aurora A, HLTF, and HDAC1, to function as a mitotic checkpoint and a tumour suppressor^1^,^2^,^3^,^4^. Notably, CHFR is able to modulate acetylation levels of histones as well as non-histone proteins like p53 and further induce *p21* expression by suppressing the HDAC1 activity^4^. Histone deacetylases (HDACs) are divided into four classes based on the sequence homology: class I (HDAC1~3 and 8), class II (HDAC4~7 and HDAC9~10), class III (SIRT1~7), and class IV (HDAC11). Class I, II, and IV are considered “classical” HDACs that utilize Zn^+^ as a cofactor and generally inhibited by trichostatin A (TSA). Meanwhile, class III HDACs, also known as sirtuins, are NAD^+^-dependent histone deacetylases and homologous to yeast Sir2 (silent information regulator 2)^5^,^6^,^7^.

SIRT1 is the most representative NAD^+^-dependent deacetylase, which belongs to the class III HDAC family^6^,^7^. SIRT1 deacetylates not only histones but also many non-histone proteins including FOXO, Ku70, p300, Rb, E2F1, NF-κB, and p53^8^,^9^,^10^. For example, SIRT1 removes an acetyl moiety from p53, resulting in the inhibition of p53-dependent cell cycle arrest and apoptosis^11^,^12^,^13^,^14^, suggesting that SIRT1 could act against p53. Consequently, through this deacetylation activity for various target substrates, SIRT1 plays a pivotal role in controlling diverse cellular processes, e.g., aging, autophagy, centrosome duplication, energy metabolism, inflammation, and tumorigenesis^15^,^16^,^17^.

Although SIRT1 is known to be regulated by several transcription factors, microRNAs, endogenous regulators such as AROS (active regulator of SIRT1) and DBC1 (deleted in breast cancer 1)^18^,^19^,^20^,^21^, and post-translational modifications, including SUMOylation^22^ and deubiquitylation^23^, the molecular machinery to regulate the expression and the activity of SIRT1 are quite complex and still remains under investigation. It has recently been reported that SIRT1 is phosphorylated by c-Jun N-terminal kinase 1 (JNK1)^24^,^25^ and subsequently degraded in a proteasome-dependent manner. Ubiquitylation is a reversible post-translational modification, which plays key roles in determining protein stability and conveying important cellular signals^26^. Therefore, it is plausible that ubiquitin-proteasome system (UPS) might be directly linked to control SIRT1 stability and function.

In the present study, we demonstrated that SIRT1 is a new target substrate of CHFR E3 Ub-ligase. CHFR binds to and ubiquitylates SIRT1, leading to its proteasomal degradation. CHFR also elevates p53 acetylation by destabilizing SIRT1, resulting in the increase of its transcriptional activity and apoptotic cell death. Especially, SIRT1 is destabilized in the presence of CHFR under oxidative stress, followed by enhanced apoptotic cell death. These results provide evidence that CHFR plays a crucial role in the regulation of SIRT1 stability and function.

## Results and Discussion

### CHFR interacts with SIRT1

**Figure 1:**
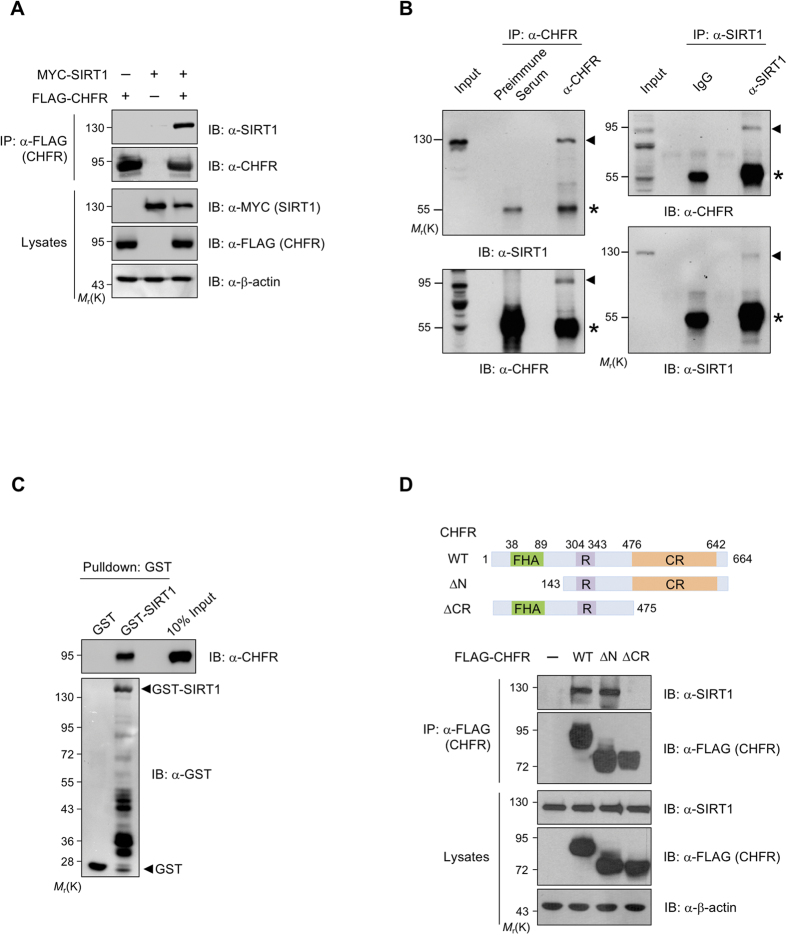
SIRT1 interacts with CHFR. (**A**) CHFR binds to SIRT1 *in vivo*. HEK293T cells were transiently transfected as indicated. Cell lysates were immunoprecipitated with anti-FLAG M2 resin and immunoblotted with indicated antibodies. (**B**) CHFR and SIRT1 are endogenously associated with each other. HeLa-*CHFR* cell lysates were immunoprecipitated with either anti-CHFR or anti-SIRT1 in combination with preimmune serum or IgG, respectively, and analyzed by immunoblotting with indicated antibodies. Asterisk and arrowhead designate IgG and immunoprecipitated target proteins, SIRT1 or CHFR, respectively. (**C**) Recombinant GST-SIRT1 and His-CHFR proteins directly interact with each other *in vitro*. Purified proteins were pulled down with glutathione-sepharose resin and immunoblotted with anti-CHFR or anti-GST antibodies. (**D**) CR domain of CHFR is required for the interaction with SIRT1. A schematic representation of CHFR with its functional domains is shown in the top panel: FHA, a forkhead-associated; R, a RING finger; and CR, a cysteine-rich domain, respectively. FLAG-CHFR DNA plasmids (WT, wild-type; ΔN, a.a. 143~664; ΔCR, a.a. 1~475, respectively) were transfected into HEK293T cells. At 24 h post-transfection, cells were treated with 2 μM MG132 for 12 h. Cell lysates were immunoprecipitated with anti-FLAG M2 resin and immunoblotted with indicated antibodies.

As CHFR is able to interact and suppress the classical HDAC^4^, we tested the possibility whether CHFR is also able to regulate another subtype of HDAC, the sirtuin family protein, SIRT1. We first performed a co-immunoprecipitation (IP) assay to determine the interaction between CHFR and SIRT1 in HEK293T cells transiently expressing FLAG-CHFR and MYC-SIRT1. When SIRT1 was immunoprecipitated with anti-FLAG M2 affinity resin, CHFR was readily detectable in the IP eluates, indicating that SIRT1 and CHFR interact with each other *in vivo* (Fig. 1A). It is worthy of note that SIRT1 protein levels were lower when SIRT1 was co-expressed with CHFR compared to when SIRT1 alone was transfected (Fig. 1A, lanes 2 and 3), suggesting that CHFR might be responsible for this SIRT1 destabilization. We also examined whether endogenous CHFR and SIRT1 could associate with each other. Since *CHFR* is often epigenetically silenced by DNA hypermethylation in most immortalized cancer cells^27^, we have generated *CHFR*-stable cells, which are identical except *CHFR* expression compared to its parental cancer cells, to mimic CHFR activation. SIRT1 was present in the immunoprecipitated eluates with anti-CHFR antibody and similarly, CHFR was present in anti-SIRT1 IP eluates, indicating that CHFR and SIRT1 bind together in HeLa-*CHFR* cells (Fig. 1B). The interaction between SIRT1 and CHFR was further validated by a GST pull-down assay using GST-SIRT1 purified from *Escherichia coli* and His-CHFR purified from Sf9 cells. His-CHFR protein was pulled down together with GST-SIRT1, but not with GST alone (Fig. 1C), indicating that the interaction between SIRT1 and CHFR is rather direct. Next, we mapped the region of CHFR required for the interaction with SIRT1. FLAG-tagged CHFR truncated mutants (ΔN, amino acids (a.a.) 143–664; ΔCR, a.a 1–475) were generated (Fig. 1D, upper panel) and tested the degree of interaction with SIRT1 in HEK293T cells in the presence of proteasome inhibitor MG132. SIRT1 was present in the IP eluates from CHFR wild-type (WT) and CHFR-ΔN, but not from CHFR-ΔCR, suggesting that the cysteine rich (CR; a.a 476–664) domain of CHFR is critical for the interaction with SIRT1 (Fig. 1D, lower panel). Taken together, CHFR binds to SIRT1 directly through its CR domain.

### CHFR ubiquitylates and promotes the proteasomal degradation of SIRT1

**Figure 2:**
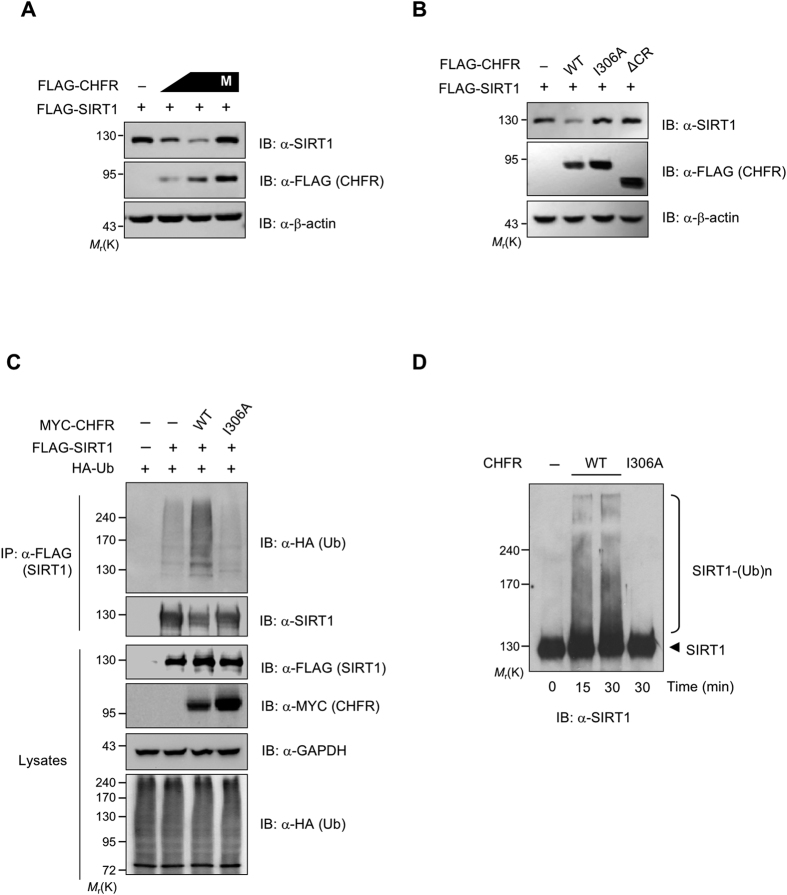
CHFR negatively regulates SIRT1. (**A**) CHFR overexpression results in the decrease of endogenous SIRT1. HeLa cells were transfected with FLAG-CHFR (0, 0.5, or 2 μg) and treated with 2 μM MG132 for 12 h as indicated. (**B**) Both an E3 Ub-ligase activity and a binding capacity to SIRT1 are necessary for CHFR to down-regulate SIRT1. FLAG-CHFR DNA plasmids encoding WT, I306A (Ile306Ala), or ΔCR mutants were transfected into HeLa cells as indicated. Cell lysates were immunoblotted with anti-FLAG and anti-SIRT1 antibodies. (**C**) CHFR ubiquitylates SIRT1 *in vivo*. HEK293T cells were transiently transfected with indicated plasmids and treated with 2 μM MG132 for 12 h. Cell lysates were immunoprecipitated with anti-FLAG M2 resin and probed with anti-HA antibody. (**D**) SIRT1 is ubiquitylated by CHFR *in vitro*. Purified SIRT1 is incubated with either CHFR WT or I306A mutant in the presence of E1, UbcH5b, ATP, ubiquitin as indicated. After *in vitro* ubiquitylation reaction, samples were analysed by immunoblotting with anti-SIRT1 antibody.

Given that SIRT1 is destabilized in the presence of CHFR as shown in Fig. 1A and they bind to each other, it is plausible that CHFR could act as a specific E3 Ub-ligase of SIRT1 to modulate its protein levels. In order to test this possibility, SIRT1 was transfected into HeLa cells, where CHFR is normally not expressed^4^, together with either mock- or CHFR-expression vector. SIRT1 protein levels were decreased in a CHFR dose-dependent manner and this reduction was blocked by the treatment of MG132 (Fig. 2A), indicating that CHFR leads to the proteasomal degradation of SIRT1. On the contrary, either E3 Ub-ligase-defective CHFR-I306A^4^ or SIRT1-binding-defective CHFR-ΔCR mutant failed to decrease SIRT1 protein levels compared to CHFR WT (Fig. 2B), suggesting that both an E3 Ub-ligase activity and a substrate binding ability of CHFR are necessary for SIRT1 destabilization.

We next sought to examine whether CHFR is able to ubiquitylate SIRT1 prior to its proteolytic degradation. HEK293T cells were transiently transfected with expression vectors encoding HA-Ub, FLAG-SIRT1, and MYC-CHFR WT or -I306A mutant, and treated with MG132. SIRT1 was heavily ubiquitylated by CHFR WT, but not by CHFR-I306A mutant (Fig. 2C). In addition, *in vitro* ubiquitylation assay under defined conditions was performed using purified E1, E2 (UbcH5b), FLAG-SIRT1, and His-CHFR (WT or I306A). CHFR WT efficiently catalyzed poly-ubiquitylation of SIRT1. However, there is no or little ubiquitylation of SIRT1 with CHFR-I306A mutant (Fig. 2D). Taken together, these results suggest that CHFR serves as a specific E3 Ub-ligase for SIRT1 ubiquitylation.

### CHFR enhances p53 acetylation and its transcriptional activity

**Figure 3:**
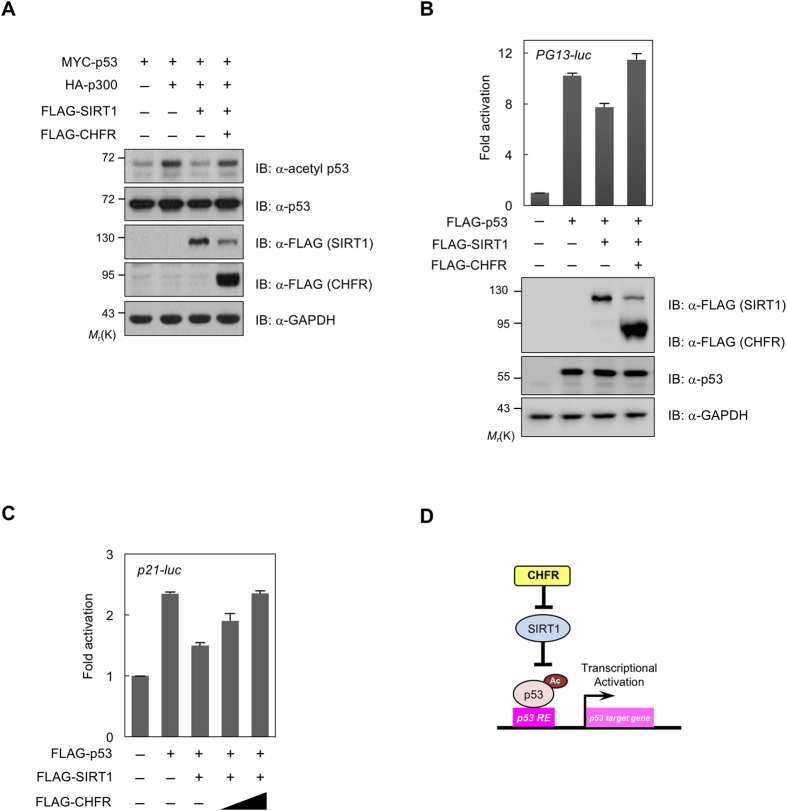
CHFR enhances the p53 transcriptional activity by destabilizing SIRT1. (**A**) p53 acetylation is restored in the presence of CHFR. MYC-p53, HA-p300, FLAG-SIRT1, and/or FLAG-CHFR were transfected into HCT116 cells and treated with 0.5 μM TSA for 6 h. Cell lysates were immunoblotted with anti-acetyl p53, anti-p53, anti-FLAG, and anti-GAPDH antibodies. (**B**–**D**) CHFR increases p53-dependent gene expression by suppressing SIRT1. HCT116 cells were transiently transfected with expression vectors for p53, SIRT1, CHFR, and β-galactosidase along with either (**B**) *PG13*- or (**C**) *p21* promoter-driven luciferase reporter gene, and treated with 0.5 μM TSA for 6 h as indicated. Luciferase activity was measured and normalized to β-galactosidase activity. Values were expressed as mean ± SEM of three independent experiments. Corresponding immunoblots are shown below (**B**). CHFR-mediated p53 transactivation is illustrated in (**D**).

As SIRT1 is able to deacetylate p53 and suppress its transcriptional activity^12^, and CHFR facilitates SIRT1 degradation, we speculated that CHFR affects p53 functions through the inhibition of the SIRT1 activity. To test this hypothesis, p53 acetylation was monitored in HCT116 cells expressing p53, p300, SIRT1, and CHFR. We have previously reported that CHFR binds to and down-regulates HDAC1, resulting in the increase of p53 acetylation^4^. To rule out such possibility that CHFR-mediated HDAC1 destabilization influences p53 acetylation, cells were then treated with TSA, a class I/II HDAC inhibitor. As expected, p53 is deacetylated by the SIRT1 introduction (Fig. 3A, lanes 2 and 3). Ectopic expression of CHFR highly elevated the levels of p53 acetylation in accordance with the reduced SIRT1 protein levels (Fig. 3A, lanes 3 and 4). These results indicate that CHFR is able to inhibit SIRT1 function not to deacetylate p53. To further validate the biological consequences of CHFR-induced SIRT1 degradation, we examined the effect of CHFR on the p53 transcriptional activity using p53 response element-containing luciferase genes, i.e., *PG13-luc* and *p21-luc*. Consistent with a previous finding, SIRT1 inhibited p53-driven gene expression and this decrease was restored by CHFR co-expression (Fig. 3B,C). These data suggest that CHFR enhances the p53 transcriptional activity by destabilizing SIRT1 (as shown in the bottom panel of Fig. 3B) and inhibiting its deacetylase activity, which are illustrated in Fig. 3D.

### CHFR is responsible for SIRT1 degradation under oxidative stress conditions

**Figure 4:**
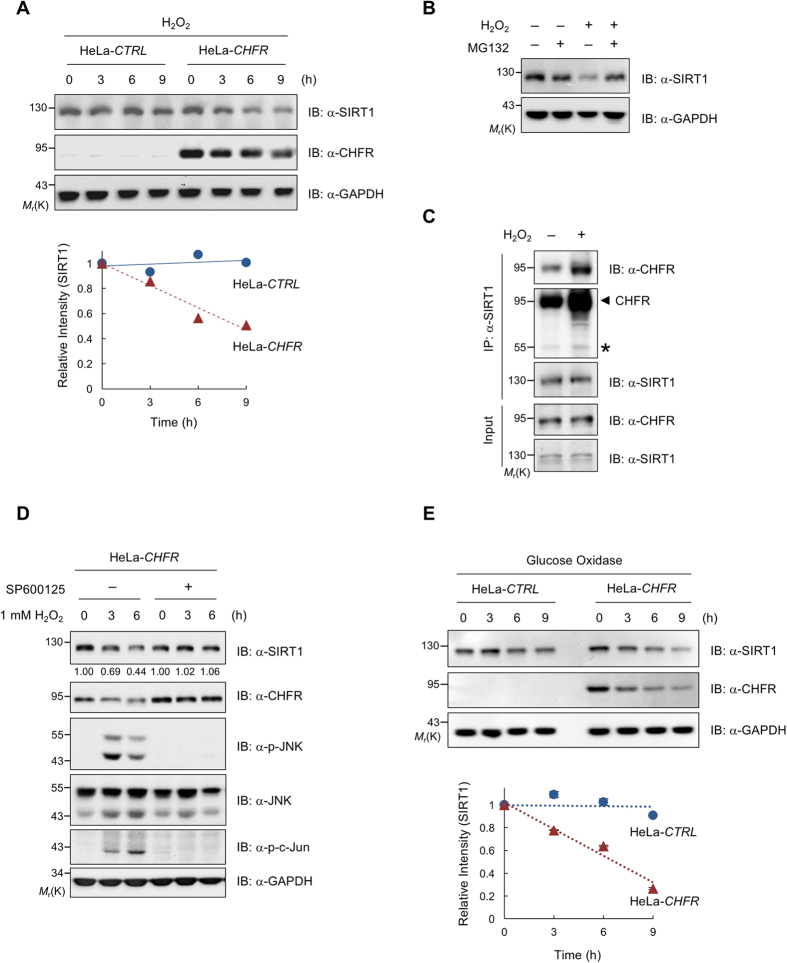
CHFR facilitates SIRT1 degradation under oxidative stress conditions. (**A**) CHFR is responsible for SIRT1 degradation upon oxidative stress. HeLa-control and –*CHFR* stable cells were treated with H_2_O_2_ as indicated and immunoblotted with anti-SIRT1, anti-CHFR, and anti-GAPDH antibodies. SIRT1 protein levels were quantitated by ImageJ and normalized to GAPDH levels. (**B**) SIRT1 is degraded via the ubiquitin-proteasome pathway upon oxidative stress. HeLa-*CHFR* stable cells were either left untreated or treated with 1 mM hydrogen peroxide for 6 h along with MG132 proteasome inhibitor as indicated. The treatment of MG132 proteasome inhibitor in cells restored SIRT1 protein levels upon oxidative stress. (**C**) CHFR interacts with SIRT1 more upon oxidative stress. HeLa-*CHFR* cell lysates treated with either mock- or 1 mM hydrogen peroxide for 6 h were immunoprecipitated with anti-SIRT1 and analyzed by immunoblotting with anti-CHFR and anti-SIRT1 antibodies. Asterisk and arrowhead designate IgG and immunoprecipitated CHFR. (**D**) HeLa-*CHFR* stable cells were treated with H_2_O_2_ in the presence or absence of SP600125 as indicated. Cell lysates were immunoblotted with anti-SIRT1, anti-CHFR, anti-phospho-JNK, anti-JNK, anti-phospho-c-Jun, and anti-GAPDH antibodies. (**E**) SIRT1 is destabilized by glucose oxidase. HeLa-control and –*CHFR* stable cells were treated with glucose oxidase as indicated and immunoblotted with anti-SIRT1, anti-CHFR, and anti-GAPDH antibodies. SIRT1 protein levels were quantitated by ImageJ and normalized to GAPDH levels.

As SIRT1 is known to be down-regulated upon oxidative stress^28^ and we have shown thus far that SIRT1 is destabilized by CHFR, we investigated whether CHFR is involved in this oxidative stress-induced SIRT1 destabilization. To test this hypothesis, we utilized HeLa-*CHFR* stable cells to assess endogenous SIRT1 protein levels and found that the treatment of 1 mM H_2_O_2_ for 6 h in cells was sufficient to decrease SIRT1 proteins (Supplementary Fig. S1A). The turn-over rate of SIRT1 in the presence of H_2_O_2_ was further determined using HeLa-control and HeLa-*CHFR* stable cells, which are identical except for expressing CHFR. Upon oxidative stress, SIRT1 was quickly destabilized only in HeLa-*CHFR* cells, indicating that CHFR is responsible for H_2_O_2_-induced SIRT1 degradation (Fig. 4A). These reduced SIRT1 protein levels were restored by the co-treatment of MG132 with H_2_O_2_, suggesting that SIRT1 is degraded by the ubiquitin-proteasome system upon oxidative stress (Fig. 4B). We have then performed the co-immunoprecipitation assay in both endogenous and transiently transfected conditions to examine whether H_2_O_2_ treatment affects the interaction between CHFR and SIRT1. Since SIRT1 is destabilized in the presence of CHFR upon H_2_O_2_ treatment, we have utilized either *CHFR*–stable cells or E3 Ub ligase-defective CHFR-I306A mutant and analysed the binding differences under the H_2_O_2_-induced oxidative stress conditions. While the binding degree of CHFR to SIRT1 was slightly increased upon H_2_O_2_ treatment, apparently, it did not seem the all-or-none differences in their interaction (Fig. 4C and Supplementary Fig. S1B). Given that SIRT1 is phosphorylated by JNK1 under oxidative stress conditions^24^,^25^, we investigated whether JNK signaling is linked to CHFR-mediated SIRT1 turn-over upon oxidative stress. When cells were treated with H_2_O_2_, the JNK pathway was activated, which was validated by the induction of phosphorylated JNK and phosphorylated c-Jun, and consequently, SIRT1 was destabilized. On the other hand, the treatment of JNK inhibitor SP600125 in cells together with H_2_O_2_ inactivated the JNK pathway and simultaneously blocked SIRT1 destabilization (Fig. 4D). Since the treatment of hydrogen peroxide is more likely to induce acute and instant damage to cells due to its quick removal by cells, we have reiterated the H_2_O_2_-driven SIRT1 destabilization under chronic oxidative stress conditions by the glucose oxidase (Gox) enzyme (Fig. 4E). Although glucose oxidase-induced chronic oxidative stress resulted in much stronger and prolonged damage to cells, consistent with the previous results shown in Fig. 4A, SIRT1 protein levels were decreased only in the presence of CHFR upon oxidative stress regardless of the type of oxidative stress triggers. Therefore, these results indicate that phosphorylated SIRT1 by JNK1 under oxidative stress conditions is destabilized by CHFR.

### CHFR promotes oxidative stress-induced cell death by destabilizing SIRT1

**Figure 5:**
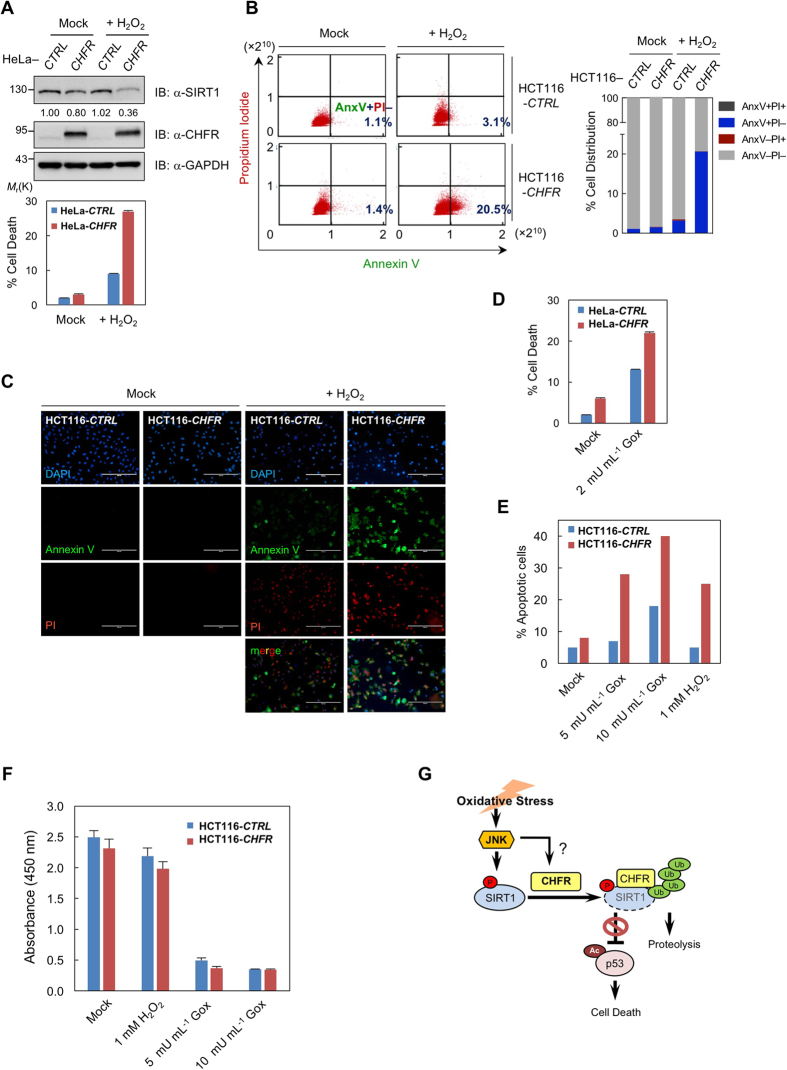
CHFR augments apoptotic cell death upon oxidative stress. (**A**) SIRT1 is destabilized in a CHFR- and H_2_O_2_-dependent manner. Cells were either left untreated or treated with H_2_O_2_ for 6 h, and immunoblotted with indicated antibodies. Annexin V/propidium iodide-based apoptotic cell death assay was performed and percent cell death values from the flow cytometry analysis were expressed as mean ± SEM of three independent experiments. (**B**–**F**) Oxidative stress-induced cell death is prominent in *CHFR*-stable cells. Cell viability was determined by annexin V/propidium iodide double staining following the treatment of either hydrogen peroxide (H_2_O_2_) or glucose oxidase (Gox) for 6 h. (**B**) Flow cytometric dot plots were sub-divided into four quadrants according to Annexin V and PI positivity and a detailed distribution of cell death events was shown. The lower left quadrant (Annexin V−/PI−) represents viable cells. The lower right quadrant (Annexin V+/PI−) was considered as early-stage apoptotic cells, the upper left (Annexin V−/PI+) as necrotic or dead cells, and the upper right quadrant (Annexin V+/PI+) as late apoptotic cells, respectively. (**C**) Annexin V−and/or PI-stained cells were monitored by the EVOS^TM^ fluorescence cell imaging system. The scale bar represents 200 μm to visualize a wider area. (**D**) Gox-induced percent total cell death, (**E**) Gox- and H_2_O_2_-induced percent apoptotic cell death, and (**F**) cell viability examined by WST-1-based CellVia assay were expressed as mean ± SEM of three independent experiments. (**G**) A proposed model for CHFR to control SIRT1 upon oxidative stress.

Given that CHFR is able to negatively regulate SIRT1 by ubiquitylation-mediated proteasomal degradation, we aimed to explore the biological outcomes of SIRT1 destabilization by CHFR, especially under oxidative stress conditions. In line with our previous results shown in Fig. 4, SIRT1 protein levels were significantly lower in H_2_O_2_-treated HeLa*-CHFR* cells compared to mock-treated cells. On the contrary, there was not much difference of SIRT1 in between mock- and H_2_O_2_-treated HeLa-control cells, indicating that CHFR is the underlying cause of SIRT1 degradation upon oxidative stress (Fig. 5A, top panel). This was further illustrated by the apoptosis assay measuring Annexin V and propidium iodide (PI) fluorescence in H_2_O_2_-treated HeLa-*CHFR* cells. Cell death was increased in HeLa-*CHFR* cells compared to control cells, and much greatly augmented by the H_2_O_2_ treatment (Fig. 5A, bottom panel). Next, we took a closer look at apoptotic events to further delineate how CHFR affects H_2_O_2_-driven cell death in HCT116-*CHFR* stable cells. Stained cells were sub-divided into four quadrants according to Annexin V and PI positivity. As cells were treated with H_2_O_2_ for 6 h, viable (Annexin V−/PI−) cells were decreased, while apoptotic (Annexin V+) and necrotic (PI+) cells were increased. Notably, H_2_O_2_-treated HCT116-*CHFR* cells showed the highest early apoptotic (Annexin V+/PI−) cell death among all tested (Fig. 5B). Moreover, Annexin V and PI fluorescence microscopy in HCT116-*CHFR* cells also revealed that CHFR is responsible for increased cell death upon oxidative stress (Fig. 5C). We have then investigated the effect of chronic oxidative stress-induced by Gox on apoptotic cell death along with SIRT1 protein levels. Cell death was much greatly augmented according to the strength of oxidative stress in *CHFR*-expressing cells (Fig. 5D,E) and similarly, SIRT1 protein levels were decreased in line with this increased cell death (Supplementary Fig. S1C). This was further validated by the cell viability assay, indicating that chronic oxidative stress by Gox leads to massive cell death (Fig. 5F).

Collectively, our data highlight that SIRT1 stability and function were negatively regulated by CHFR-mediated ubiquitylation and subsequent proteolysis. The inhibition of SIRT1 in human cancer cells by CHFR expression leads to elevated acetylation of p53 and simultaneous trans-activation of p53-driven target genes to elicit apoptosis in response to oxidative stress (Fig. 5G).

It is worthy of note that not only SIRT1 but also CHFR were destabilized by H_2_O_2_ in *CHFR*-stable cells. Since CHFR is known to be regulated by its own auto-ubiquitylation activity^29^, it would be of interest to study whether the E3 Ub-ligase activity of CHFR is controlled by the JNK signaling pathway. As both CHFR and SIRT1 have been implicated in cell cycle control and tumorigenesis, it would also be of particular interest to investigate how and when CHFR and/or SIRT1 respond to diverse cellular stresses during tumour progression. Here, we aimed to add a new line of evidence how CHFR contributes to tumour suppression. Especially, we have shown that CHFR is able to suppress not only HDAC1- class I HDAC^4^, but also SIRT1- class III HDAC. CHFR is often epigenetically inactivated in various cancer cells^27^, and reduced CHFR expression in normal cells leads to tumorigenic phenotypes^30^. Accordingly, such CHFR malfunction may lead to SIRT1 stabilization, which in turn represses p53 and other tumour suppressors to accelerate tumour initiation and metastasis. We have shown thus far that CHFR elevates the p53 activity by destabilizing SIRT1 when CHFR was re-introduced into cancer cells. Since p53 is widely regarded as “the guardian of genome”^31^,^32^, it is plausible that CHFR becomes a part of the watchman to keep cells under surveillance. This reinforces the role of CHFR as a tumour suppressor. CHFR also acts as a cell cycle checkpoint^33^, therefore, CHFR helps to maintain the cellular integrity against harmful stimuli and the threshold for apoptosis and cell senescence.

## Methods

### Cell culture, Transfection, and Reagents

HCT116, HeLa, and HEK293T cells were cultured in Dulbecco’s modified Eagle’s medium supplemented with 100 U ml^−1^ penicillin, 100 μg ml^−1^ streptomycin, and 10% FBS (Gibco) at 37 °C in a humidified 5% CO_2_ condition. Transient and stable transfections were carried out using either lipofectamine 2000 (Invitrogen) or polyethylenimine (Sigma) according to the manufacturer’s instructions. Following chemical reagents used in the study were obtained from Sigma or otherwise stated: TSA, hydrogen peroxide, glucose oxidase, and SP600125.

### Immunoprecipitation and Immunoblotting

For immunoprecipitation, cells were lysed in TNET buffer (20 mM Tris-HCl [pH 7.5], 150 mM NaCl, 0.1 mM EDTA, and 0.2% Triton X-100) and 1x protease inhibitor cocktail (Roche). Cell lysates were incubated with anti-FLAG M2 affinity resin (Sigma) for 2 h at 4 °C. Resins were collected by centrifugation and washed three times with TNET buffer. Bound proteins were eluted, resolved by SDS-PAGE, and immunoblotted with appropriate antibodies. The following antibodies were used: anti-SIRT1, anti-MYC, anti-GST, anti-GAPDH, anti-HA, and anti-p53 (Santa Cruz Biotechnology); anti-acetyl p53 (Millipore); anti-FLAG and anti-β-actin (Sigma); anti-Xpress (Invitrogen); peroxidase-conjugated AffiniPure goat anti-rabbit and anti-mouse IgGs (Jackson ImmunoResearch); anti-CHFR antiserum was raised against a recombinant His-CHFR. Relative protein levels in the immunoblot figures were quantitated by ImageJ and normalized to either β-actin or GAPDH levels. Values are plotted as the mean ± SEM of at least three independent experiments.

### GST pull-down assay

GST-SIRT1 was purified from *Escherichia coli* and His-CHFR was purified from Sf9 insect cells. GST-SIRT1 (1 μg) and His-CHFR (1 μg) were incubated with Glutathione Sepharose 4 Fast Flow (GE Healthcare) for 1 h at 4 °C. After incubation, bound proteins were eluted, resolved by SDS-PAGE, and analysed by immunoblotting with anti-CHFR and anti-GST antibodies.

### Ubiquitylation assay

For the *in vitro* ubiquitylation assay, FLAG-SIRT1 protein (0.3 μg) purified from HEK293T cells was incubated with E1 (0.2 μg), UbcH5b (0.2 μg), Ubiquitin (2 μg), CHFR (1 μg), and ATP-regenerating system (50 mM Tris-HCl [pH 7.5], 5 mM MgCl_2_, 10 mM creatine phosphate, 5 U ml^−1^ of phosphocreatine kinase, and 5 mM ATP) at 37 °C for indicated times. For the *in vivo* ubiquitylation assay, cells were transfected with appropriate expression vectors and treated with 2 μM MG132 (A.G. Scientific) for 12 h. Cell lysates were incubated with anti-FLAG M2 affinity resin (Sigma). After incubation, bound proteins were eluted and analysed by immunoblotting.

### Reporter assay

HCT116 cells were transfected with indicated plasmids with β-gal constructs and treated with 0.5 μM TSA for 6 h before harvest. Luciferase activity was measured in a luminometer with a luciferase system (Promega) and normalized to β-galactosidase activity. Values were expressed as mean ± SEM from three independent experiments.

### Apoptosis assay

Cells were treated with either hydrogen peroxide or glucose oxidase for indicated times to induce oxidative stress, stained with either Alexa Fluor^®^ 488 Annexin V/Dead Cell Apoptosis Kit (Molecular Probes) or FITC Annexin V Apoptosis Detection Kit (BD Biosciences) according to manufacturers’ instructions, and analysed using the TaLi^®^ image-based cytometer (Invitrogen) or visualized under the EVOS^TM^ cell imaging system (Thermo Fisher Scientific). DAPI was used to counterstain the nuclei.

### Cell viability assay

Cells were seeded at a density of 10^4^ cells/well in 100 μL of culture medium in a 96-well plate and treated with either hydrogen peroxide or glucose oxidase for 6 h to induce oxidative stress. Cells were treated with 10 μL of CellVia (water-soluble tetrazolium salt, WST-1; Young In Frontier) and incubated for an additional 2 h at 37 °C. Cell viability was measured using a multiwell microplate reader at a wavelength of 450 nm along with a reference wavelength of 650 nm. The same volume of culture medium plus CellVia reagent in the absence of cells were used as a blank control to subtract the background absorbance. Values were expressed as mean ± SEM from three independent experiments.

## Additional Information

**How to cite this article**: Kim, M. *et al*. CHFR negatively regulates SIRT1 activity upon oxidative stress. *Sci. Rep.* **6**, 37578; doi: 10.1038/srep37578 (2016).

**Publisher’s note:** Springer Nature remains neutral with regard to jurisdictional claims in published maps and institutional affiliations.

## Supplementary Material

Supplementary Figure
