# Supplementary material for: CHFR negatively regulates SIRT1 activity upon oxidative stress
Source: Sci Rep. 2016 Nov 24;6:37578. doi: 10.1038/srep37578 (PMC5121620; doi:10.1038/srep37578)
Supplement: Supplementary Figure [file srep37578-s1.pdf]

CHFR negatively regulates SIRT1 activity upon oxidative stress.

Myungjin Kim, Young Eun Kwon, Jae Oh Song, Sung Jun Bae, and Jae Hong Seol*

School of Biological Sciences, Research Institute of Basic Sciences, IMCB, Seoul National University, Seoul 08826, Korea

Manuscript for *Scientific Reports*

*To whom correspondence should be addressed.

Tel: +82-2-880-6688; Fax: +82-2-872-1993

E-mail: jhseol@snu.ac.kr (JHS)

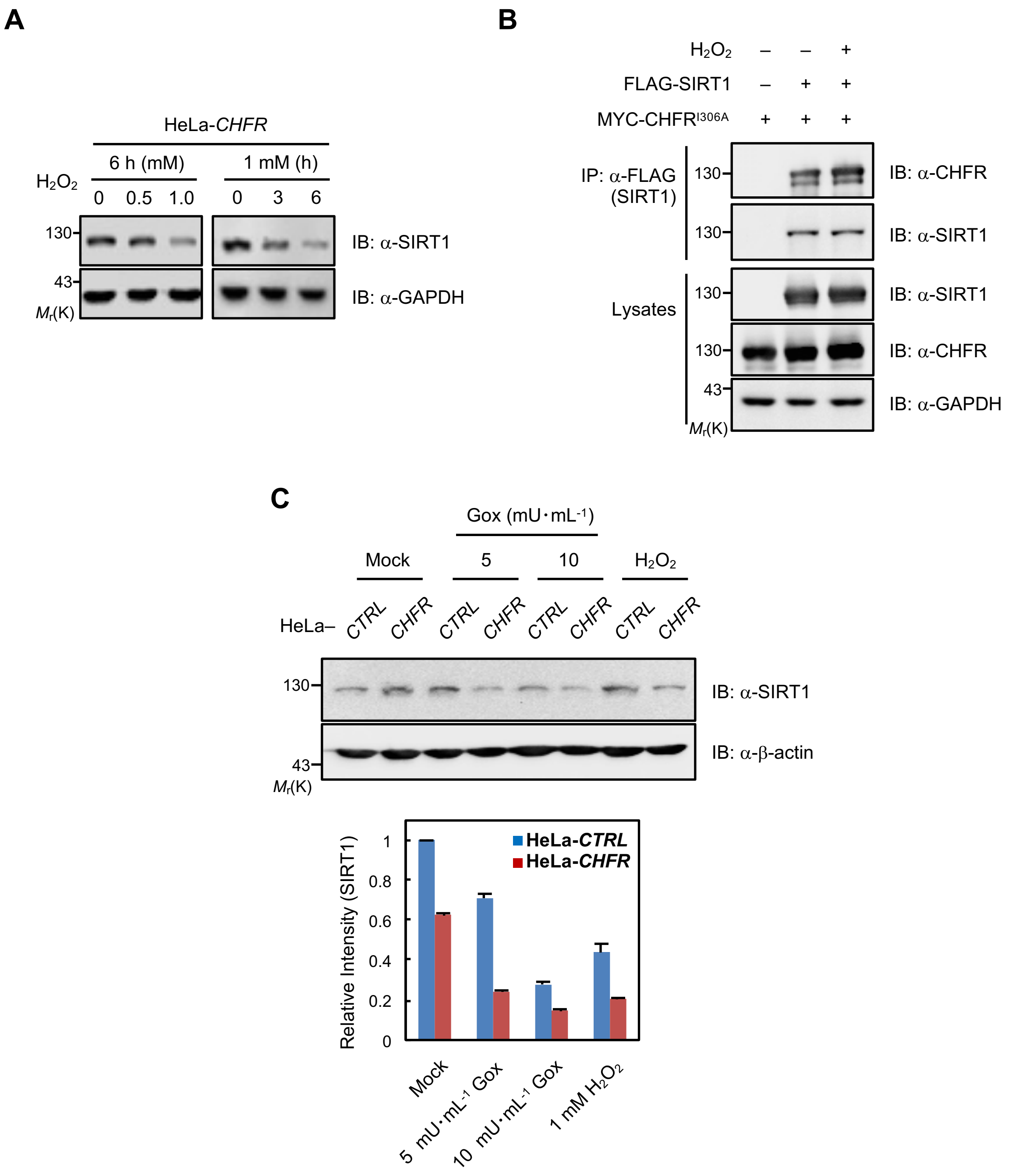

Figure S1. SIRT1 is destabilized upon oxidative stress: in conjunction with Figure 4 and 5. (A) SIRT1 is destabilized by H_2O_2. Effects of H_2O_2 treatment on SIRT1 protein levels were assessed in HeLa-*CHFR* cells in a dose- and time-dependent manner as indicated. (B) CHFR interacts with SIRT1 more upon oxidative stress. HEK293T cells were transiently transfected as indicated and treated with either mock- or 1 mM hydrogen peroxide for 6 h. Cell lysates were immunoprecipitated with anti-FLAG M2 resin and immunoblotted with indicated antibodies. (C) SIRT1 is destabilized upon oxidative stress. HeLa-*CHFR* stable cells were treated for 6 h with following oxidative stress inducers: mock, 5 mU/mL or 10 mU/mL glucose oxidase, 1 mM hydrogen peroxide. Cell lysates were immunoblotted with indicated antibodies. SIRT1 protein levels were quantitated by ImageJ and normalized to β-actin levels.
